# Waves in spatio-spectral and -temporal coherence of evolving ultra-intense twin beams

**DOI:** 10.1038/s41598-019-39482-x

**Published:** 2019-03-12

**Authors:** Jan Peřina

**Affiliations:** Joint Laboratory of Optics of Palacký University and Institute of Physics of the Czech Academy of Sciences, Faculty of Science, Palacký University, 17. listopadu 12, 77146 Olomouc, Czech Republic

## Abstract

Waves in the spatio-spectral and -temporal coherence of evolving ultra-intense twin beams are predicted: Twin beams with low intensities attain maximal coherence in the beam center until certain threshold intensity is reached. Then the area of maximal coherence moves with increasing intensity from the beam center towards its edges leaving the beam center with low coherence (the first coherence wave). For even larger intensities, a new coherence maximum is gradually built in the beam center with the increasing intensity and, later, it again moves towards the beam edges forming the second coherence wave. Rotationally-symmetric twin beams are analyzed within a three-dimensional model that couples spectral and spatial degrees of freedom. Relation between the twin-beam coherence and its local density of modes during the nonlinear evolution is discussed.

## Introduction

Parametric down-conversion, in parallel with its applications in classical nonlinear optics^1^, nowadays serves as a common source of three types of highly nonclassical states^2^: states of individual entangled photon pairs, intense twin beams composed of macroscopic numbers of photon pairs and states with squeezed phase and photon-number fluctuations (squeezed, sub-Poissonian light). The states of individual photon pairs have been intensively studied for already more than 40 years^3^. Several milestones in modern physics were reached with their help including the experimental demonstration of the Bell-inequalities violation^4^ and experimental teleportation of the polarization state of a photon^5^. Also the squeezed states that are provided by the degenerate variant of parametric down-conversion in resonators have been investigated extensively^6,7^. This has resulted in the construction of sources of squeezed light that exhibit the reduction of vacuum fluctuations better than 15 dB^8^. Both these types of states are at present, apart from their applications in ultra-precise metrology, considered as promising for quantum-information processing, especially in connection with optical communications. On the other hand, much lower attention has been paid to *intense twin beams* (TWB) that emerge in parametric down-conversion in the running-wave configuration and contain macroscopic, i.e. large, numbers of photon pairs^9–19^. Their sub-shot-noise intensity correlations between the signal and idler beams that constitute a TWB have been demonstrated^20–25^ and even exploited for sub-shot-noise imaging^26^. Also tight spatial correlations between the signal and idler beams that originate in phase matching of the nonlinear interaction have been analyzed in different configurations^27–31^. Applications of TWBs aiming at improving the experimental precision have been suggested in spectroscopy^32^ and quantum interferometry^33^. Also TWBs with squeezed spectrally broad-band phase fluctuations have been suggested^34^.

The generated TWBs are naturally highly multi-mode, both in their spectra and spatial transverse modes^9,30,35–37^. The need to generate TWBs composed of ideally only one spatio-spectral mode triggered investigations of both spectral and spatial coherence of intense TWBs^29,30,36^. An increase of both spectral and spatial coherence with an increasing TWB intensity has been experimentally confirmed for the beams with lower intensities^30^. On the other hand, it has been observed that, for the sufficiently intense TWBs, degradation of coherence occurs^36^. This degradation originates in considerable depletion of the pump beam and we speak about *ultra-intense twin beams* in this case. This behavior has been confirmed by direct numerical solution of the nonlinear Maxwell equations^36^. A model of intense parametric down-conversion based upon the decomposition of optical beams into the Schmidt dual modes shed more light into the dynamics of formation of such ultra-intense TWBs by considering the evolution of individual modes inside the interacting pump, signal and idler beams^38,39^. It revealed that an increase (decrease) of coherence of a TWB results from the reduction (increase) of the number of effectively populated modes inside the TWB, which occurs as a consequence of the dynamics of individual modes’ triplets each being composed of one mode in the signal, idler and pump beam. The model presented in ref.^39^ has shown that the maximal coherence quantified by *spatially and spectrally averaged* intensity correlation functions attains its maximum for certain TWB intensity (that is generated by appropriate pump power for a given crystal length). For this intensity, the TWB is composed of the lowest possible number of modes, that is considerably lower than the number of modes constituting a weak TWB. Multiple coherence maxima occurring in the averaged intensity correlation functions are predicted for even higher pump powers^39^. For such beams, also photon-number statistics were investigated^40^. Multi-mode thermal photon-number statistics, which are a consequence of the initial spontaneous process, were experimentally verified^35^ for lower pump powers. In the regime with pump depletion, ultra-intense TWBs containing both chaotic (multi-mode thermal) and coherent components are predicted^40^. The generation of ultra-intense TWBs in poled nonlinear crystals was also addressed^41^.

**Figure 1:**
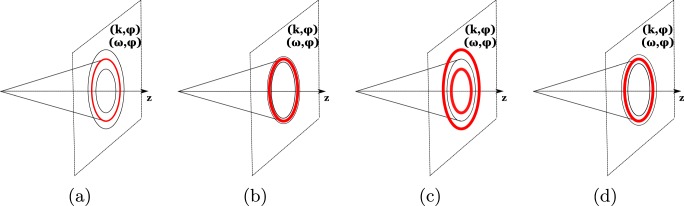
A signal (or idler) beam in its transverse wave-vector plane at four subsequent stages during its evolution in a sufficiently-long nonlinear crystal: (**a**) broad weak beam with only weakly developed coherence in the central part, (**b**) narrow stronger beam with well developed coherence in the central part, (**c**) broad strong beam with well developed coherence at the edges, and (**d**) narrow even stronger beam with well developed coherence in the central part. The degree of coherence is quantitatively indicated by the widths of red ellipses. The first coherence wave in the TWB evolution occurs between stages (**b**) and (**c**). The second coherence wave begins to propagate from stage (**d**). Qualitatively the same evolution occurs also in the plane in which the radial transverse wave-vector *k* is substituted by frequency *ω*.

These recent findings point out at reach physical behavior of ultra-intense TWBs. Here, we extend these investigations by addressing coherence of intense and ultra-intense TWBs inside their spatio-spectral as well as spatio-temporal profiles. We reveal that the coherence of TWBs considerably varies across these profiles and it also qualitatively changes with the varying TWB intensity: There exist TWBs with maximal coherence in the center of their profiles, similarly as TWBs exhibiting coherence maxima at the edges of their profiles. Different kinds of such TWBs naturally occur when we follow the evolution of a TWB from the vacuum along a sufficiently long nonlinear crystal through which an intense ’source’ pump beam propagates. There occur two qualitatively different stages in the evolution of the TWB, as schematically illustrated in Fig. 1. Concentrating on the properties of the rotationally-symmetric signal (or similarly idler) beam in its transverse wave-vector plane (experimentally reached in the far field) we observe the following evolution: The initial weak broad signal beam endowed with little coherence mainly in its center [see Fig. 1(a)] gradually takes energy from the pump pulse dominantly due to stimulated emission. This leads to narrowing of the signal beam as the greatest amount of energy comes to the area around the beam center. Simultaneously, the stimulated emission gradually improves coherence of the signal beam mainly in this area, as shown in Fig. 1(b). Due to the complex mode evolution in the nonlinear process, as explained below, at certain moment of the TWB evolution, narrowing of the signal beam terminates and the beam broadens. This indicates termination of the first stage of TWB coherence evolution and beginning of the second stage. At this moment, the coherence induced by the previous evolution of the signal beam does not deteriorate, but, roughly speaking, it starts to move from the beam center towards its edges. The area of maximal coherence reaches the beam edges for intensities at which the signal beam becomes the broadest [see Fig. 1(c)] and then it ’disappears’ in the beam tails with negligible intensity. This means the end of *the first coherence wave*. In the subsequent period of the signal-beam narrowing, a new coherence peak is gradually formed in the beam center. The maximal coherence in this peak is reached when the signal beam attains its narrowest shape [see Fig. 1(d)]. Then, the area of maximal coherence starts to move from the beam center towards its edges forming *the second coherence wave*. In a real three-dimensional (3D) rotationally-symmetric TWB the discussed behavior in the transverse wave-vector plane is tightly accompanied by qualitatively the same behavior in the spectral (as well as temporal) space. These 2D coherence waves that originate in the internal dynamics of a TWB represent the main finding of the article. In the article, we analyze the evolution of a 3D TWB in detail. Using the developed model we also interpret the TWB coherence in terms of the local densities of beam modes that considerably change during the nonlinear evolution.

To understand this behavior, the determination of *spatially and spectrally resolved* correlation functions is needed. This has required qualitative generalization of the model of ultra-intense TWBs of ref.^39^ that provides only the spatially and spectrally averaged correlation functions in which these waves are not visible. In the article, we not only provide this generalization we also incorporate into the model the mutual coupling between the spectral and radial wave-vector degrees of freedom in a rotationally-symmetric TWB. This allows us to analyze also spectrally non-degenerate TWBs.

At the end of Introduction, we would like to note that the theory developed here for parametric down-conversion^1^ can be modified such that nonlinear resonant interactions involving four-wave mixing in cold atomic ensembles^42–44^ are described. Compared to parametric down-conversion, much higher effective nonlinear coupling constants occur for resonant interactions and also parameters of the generated optical beams are more appropriate when subsequent absorptions by atoms are considered. High values of sub-shot-noise intensity correlations between the beams were observed in such systems^45–47^. Combinations of several nonlinear resonant processes were also used to generate more complex multi-mode states endowed with quantum correlations^48–51^. Whereas the properties of intense TWBs arising in parametric interactions are more appropriate for metrology applications, intense TWBs and their generalizations reached in (multiple) resonant interactions in atomic ensembles are suitable for implementations of various quantum-information protocols. Metrology applications whose measurement uncertainties are suppressed below the classical limit^20,34^ due to the TWB sub-shot-noise intensity correlations may further profit from additional stability due to the large number of photon pairs inside intense TWBs^32,33^.

The paper is organized as follows. The suggested model is described in section **Three-dimensional model of an intense twin beam**. Quantities that characterize an intense TWB including its coherence are determined in section **Physical quantities characterizing twin beams**. TWB intensities, numbers of modes and intensity profiles are discussed in section **Twin-beam intensities, intensity profiles and numbers of internal modes**. TWB coherence as it evolves with the increasing pump power and typical profiles of intensity auto- and cross-correlation functions are analyzed in section **Spatial, spectral and temporal coherence of twin beams, coherence waves**. Section **Twin-beam coherence and density of modes** is devoted to the relation between the TWB coherence and its density of modes. Evolution of the coherence of a TWB propagating along a long crystal is described in section **Coherence of a twin beam propagating along the crystal**. Section **Conclusions** summarizes the main findings.

## Three-Dimensional Model of an Intense Twin Beam

Parametric down-conversion as the second-order nonlinear process is characterized by tensor *χ*^(2)^ of second-order nonlinear susceptibility that mediates an effective nonlinear interaction among the signal (index s), idler (i) and pump (p) beams. Assuming propagation of these beams along the *z* axis an interaction momentum operator ${\hat{G}}_{{\rm{int}}}$ appropriate for this process is written as follows^52,53^:

1 $${\hat{G}}_{{\rm{int}}}(z)=2{\varepsilon }_{0}\int dxdy{\int }_{-\infty }^{\infty }dt[{\chi }^{\mathrm{(2)}}:{E}_{{\rm{p}}}^{(+)}({\bf{r}},t){\hat{E}}_{{\rm{s}}}^{(-)}({\bf{r}},t){\hat{E}}_{{\rm{i}}}^{(-)}({\bf{r}},t)+{\rm{H}}\mathrm{.}{\rm{c}}.]$$

and **r** = (*x*, *y*, *z*). The positive-frequency part of a classical pump electric-field amplitude is denoted as ${E}_{{\rm{p}}}^{(+)}$ similarly as the negative-frequency part of a signal [idler] electric-field operator amplitude is referred as ${\hat{E}}_{{\rm{s}}}^{(-)}$ [${\hat{E}}_{{\rm{i}}}^{(-)}$]. Symbol H.c. replaces the Hermitian conjugated term, *ε*_0_ means the permittivity of vacuum, and symbol: shorthands the *χ*^(2)^ tensor with respect to its three indices.

We decompose the electric-field amplitudes ${E}_{a}^{(+)}({\bf{r}},t)$ [${E}_{a}^{(+)}({\bf{r}},t)={E}_{a}^{(-)\ast }({\bf{r}},t)$] of the interacting beams into harmonic plane waves with wave vectors **k**_*a*_(*ω*_*a*_) and frequencies *ω*_*a*_:

2 $${E}_{a}^{(+)}({\bf{r}},t)=\frac{1}{{\sqrt{2\pi }}^{3}c}\int d{{\bf{k}}}_{a}^{\perp }\int d{\omega }_{a}{E}_{a}^{(+)}({{\bf{k}}}_{a}^{\perp },{\omega }_{a})\exp [i{{\bf{k}}}_{a}({\omega }_{a}){\bf{r}}-i{\omega }_{a}t],\,a={\rm{p}},{\rm{s}},{\rm{i}};$$

*c* is the speed of light in vacuum. We invoke the paraxial approximation for the propagating beams and so we identify the harmonic plane waves by the transverse parts ${{\bf{k}}}_{a}^{\perp }$ of their wave vectors and frequencies *ω*_*a*_.

An intense pump beam with amplitude *ξ*_p_, central frequency ${\omega }_{{\rm{p}}}^{0}$, propagating along the *z* axis and having Gaussian profiles both in the transverse plane (beam radius *w*_p_) and temporal spectrum (pulse duration *τ*_p_) is assumed to impinge on a nonlinear crystal of length *L*:

3 $${E}_{{\rm{p}}}^{(+)}({{\bf{k}}}_{{\rm{p}}}^{\perp },{\omega }_{{\rm{p}}})={\xi }_{p}\frac{\sqrt{{\tau }_{{\rm{p}}}}{w}_{{\rm{p}}}}{{\mathrm{(2}\pi )}^{\mathrm{3/4}}}\exp [-\frac{{w}_{{\rm{p}}}^{2}({k}_{{\rm{p}},x}^{2}+{k}_{{\rm{p}},y}^{2})}{4}]\exp [-\frac{{\tau }_{{\rm{p}}}^{2}{({\omega }_{{\rm{p}}}-{\omega }_{{\rm{p}}}^{0})}^{2}}{4}].$$

Knowing the pump-beam power *P* and its repetition rate *f*, we have ${\xi }_{{\rm{p}}}=\sqrt{P{\omega }_{{\rm{p}}}^{0}}/\sqrt{{\varepsilon }_{0}{c}^{2}{k}_{{\rm{p}}}\,f}$ for the incident pump-beam amplitude. In Eq. (3), we assume that the pump-beam transverse profile does not change along the *z* axis if the nonlinear interaction is neglected. This ‘cylindrical approximation’ means that the crystal length has to be smaller than the Rayleigh range of the used pump Gaussian beam.

The complex nonlinear dynamics of three interacting beams can successfully be treated when we introduce suitable spatio-spectral modes in each beam that approximately ’diagonalize’ the interaction among three beams and that replace the usual harmonic plane waves^39,40^. Such spatio-spectral modes’ triplets can be found by first considering the Schmidt dual modes in the signal and idler beam revealed by the Schmidt decomposition^54^ of a two-photon spatio-spectral amplitude Φ_si_ of a state containing one photon pair (for details, see below). A pump-beam mode that corresponds to a given pair of signal and idler modes and belongs to a common modes’ triplet is obtained by considering one nonlinear quantum event with simultaneous annihilation of a signal and an idler photon and creation of a pump photon in a classical fashion^40^: A signal photon with its classical electric-field amplitude in the form of a given mode function together with its idler twin with the classical electric-field amplitude given by its mode function undergo classical nonlinear interaction that provides, according to the rules of classical nonlinear optics, the profile of classical electric-field amplitude of a pump photon. This physically motivated approach has two drawbacks. First, the obtained pump-mode profiles are not normalized to unity, as needed in any quantum model. Second, the pump-mode profiles determined for different Schmidt dual signal and idler modes are not exactly orthogonal. The first drawback is easily removed by re-normalizing the pump modes to unity^40^ and simultaneously changing the corresponding nonlinear effective coupling constant for the corresponding modes’ triplet. As for the second drawback, numerical analysis of non-orthogonality of pump modes reveals that the largest overlap is between the nearest neighbors and drops down fast as the distance between modes (expressed by the difference of modes index numbers) grows. According to our experience with down-conversion pumped by pulses with duration in units of ps, the greatest overlaps of pump mode functions of the nearest neighbors are lower than 15%, the overlaps for the first couple of more distant modes are only in units of %, and the remaining overlaps are practically zero. This behavior arises from the typical oscillatory behavior of pump-mode profiles, a *q*th 1D mode has 2*q* + 1 peaks in its intensity profile for *q* = 0, 1, …. We note that a *q*th 1D signal (or idler) mode has only *q* + 1 peaks in its intensity profile. Omission of these overlaps is the essence of the used approximation. It leads to the picture of many independent modes’ triplets with their own internal dynamics. As there exists no parallel quantum model of intense TWBs, validity and applicability of the used model can only be judged by comparing its predictions with the experiment and classical numerical simulations that mimic quantum behavior. The model was successfully used to interpret the properties of experimental TWBs in ref.^38^. On the other hand, it was shown in ref.^36^ that the properties of experimental TWBs accord with the results of classical numerical simulations based on the solution of the nonlinear Maxwell equations. In our opinion, highly multi-mode character of intense TWBs importantly contributes to the applicability of this approximation.

Contrary to the case of spectrally degenerate TWBs analyzed in refs.^39,40^, the consideration of spectrally non-degenerate TWBs requires a general procedure for obtaining the dual Schmidt modes. The procedure begins with the determination of the state |*ψ*_si_〉 of one photon pair leaving the nonlinear crystal. For the crystal that extends from *z* = −*L* to *z* = 0 the state |*ψ*_si_〉 is obtained as a first-order perturbation solution of the Schrödinger equation assuming the incident vacuum state |vac_si_〉 in the signal and idler beams:

4 $$|{\psi }_{{\rm{si}}}\rangle =\frac{i}{\hslash }{\int }_{-L}^{0}dz{\hat{G}}_{{\rm{int}}}(z)|{{\rm{vac}}}_{{\rm{si}}}\rangle =\int d{{\bf{k}}}_{{\rm{s}}}^{\perp }\int d{\omega }_{{\rm{s}}}\int d{{\bf{k}}}_{{\rm{i}}}^{\perp }\int d{\omega }_{{\rm{i}}}{{\rm{\Phi }}}_{{\rm{si}}}({{\bf{k}}}_{{\rm{s}}}^{\perp },{\omega }_{{\rm{s}}},{{\bf{k}}}_{{\rm{i}}}^{\perp },{\omega }_{{\rm{i}}}){\hat{a}}_{{\rm{s}}}^{\dagger }({{\bf{k}}}_{{\rm{s}}}^{\perp },{\omega }_{{\rm{s}}}){\hat{a}}_{{\rm{i}}}^{\dagger }({{\bf{k}}}_{{\rm{i}}}^{\perp },{\omega }_{{\rm{i}}})|{{\rm{vac}}}_{{\rm{si}}}\rangle \mathrm{.}$$

In Eq. (4), the operator ${\hat{a}}_{a}^{\dagger }({{\bf{k}}}_{a}^{\perp },{\omega }_{a})$ creates a photon in mode $({{\bf{k}}}_{a}^{\perp },{\omega }_{a})$ and $\hslash$ stands for the reduced Planck constant. The explicit formula for the two-photon amplitude Φ_si_ introduced in Eq. (4) can be found in ref.^55^.

In the model, we assume rotational symmetry (with respect to the *z* axis along which the pump beam propagates) of the analyzed system. This means that the model is suitable for type-I parametric down-conversion in collinear or not-to-far-from collinear geometry for sufficiently wide pump-beam transverse profiles, as discussed in detail in ref.^12^. This symmetry implies that the general two-photon amplitude Φ_si_ depends only on the difference *φ*_s_ − *φ*_i_ of azimuthal angles of the signal and idler modes [${{\bf{k}}}_{a}^{\perp }\equiv ({k}_{a}^{\perp }\,\sin ({\phi }_{a}),{k}_{a}^{\perp }\,\cos ({\phi }_{a}))$, *a* = s, i]:

5 $${{\rm{\Phi }}}_{{\rm{si}}}({{\bf{k}}}_{{\rm{s}}}^{\perp },{\omega }_{{\rm{s}}},{{\bf{k}}}_{{\rm{i}}}^{\perp },{\omega }_{{\rm{i}}})=\frac{1}{2\pi }\sum _{m=-\infty }^{\infty }{{\rm{\Phi }}}_{m}({k}_{{\rm{s}}}^{\perp },{\omega }_{{\rm{s}}},{k}_{{\rm{i}}}^{\perp },{\omega }_{{\rm{i}}})\exp [im({\phi }_{{\rm{s}}}-{\phi }_{{\rm{i}}}\mathrm{)].}$$

The azimuthal functions Φ_*m*_ occurring in the azimuthal decomposition (5) are determined along the formula:

6 $${{\rm{\Phi }}}_{m}({k}_{{\rm{s}}}^{\perp },{\omega }_{{\rm{s}}},{k}_{{\rm{i}}}^{\perp },{\omega }_{{\rm{i}}})=\sqrt{{k}_{{\rm{s}}}^{\perp }{k}_{{\rm{i}}}^{\perp }}{\int }_{0}^{2\pi }d({\phi }_{{\rm{s}}}-{\phi }_{{\rm{i}}}){{\rm{\Phi }}}_{{\rm{si}}}({{\bf{k}}}_{{\rm{s}}}^{\perp },{\omega }_{{\rm{s}}},{{\bf{k}}}_{{\rm{i}}}^{\perp },{\omega }_{{\rm{i}}})\exp [\,-\,im({\phi }_{{\rm{s}}}-{\phi }_{{\rm{i}}}\mathrm{)].}$$

**Figure 2:**
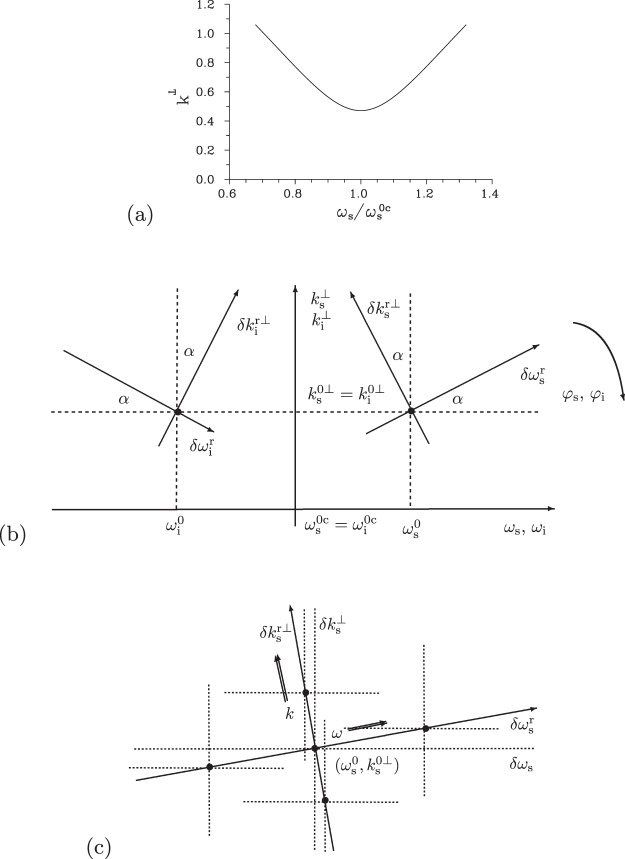
(**a**) Phase matching curve ${k}^{\perp }({\omega }_{{\rm{s}}})\equiv {k}_{{\rm{s}}}^{\perp }({\omega }_{{\rm{s}}})={k}_{{\rm{i}}}^{\perp }({\omega }_{{\rm{p}}}^{0}-{\omega }_{{\rm{s}}})$ as a function of normalized signal frequency ${\omega }_{{\rm{s}}}/{\omega }_{{\rm{s}}}^{0{\rm{c}}}$; ${\omega }_{{\rm{s}}}^{0{\rm{c}}}={\omega }_{{\rm{p}}}^{0}\mathrm{/2}$. (**b**) Sketch of the used signal- and idler-beam coordinate systems. Variables $\delta {k}^{{\rm{r}}\perp }$ and *δω*^r^ belong to the coordinate systems rotated by angle *α*, whereas variables *k*^⊥^ and *ω* are defined in the original coordinate system. The azimuthal angles *φ* are identical in both systems. The rotated signal [idler] coordinate system is centered at position $({k}_{{\rm{s}}}^{0\perp },{\omega }_{{\rm{s}}}^{0})$ [$({k}_{{\rm{i}}}^{0\perp },{\omega }_{{\rm{i}}}^{0})$] where ${k}_{{\rm{s}}}^{0\perp }={k}_{{\rm{i}}}^{0\perp }$ and ${\omega }_{{\rm{s}}}^{0}+{\omega }_{{\rm{i}}}^{0}={\omega }_{{\rm{p}}}^{0}$. (**c**) Sketch of the signal-beam rotated coordinate system showing two perpendicular systems of cuts positioned along *ω*^r^ and *k*^r⊥^ directions. At each point indicated by a bullet, three orthogonal cuts parallel to the axes *k*^⊥^, *ω* and *φ* are investigated.

To arrive at a Schmidt-mode decomposition^56,57^ of the azimuthal two-photon amplitudes Φ_*m*_ that admits factorization in its variables as a suitable approximation, we rotate the orthogonal coordinate systems $({k}_{a}^{\perp },{\omega }_{a})$ around the central points $({k}_{a}^{0\perp },{\omega }_{a}^{0})$ that are chosen in the centers of radial wave-vector and spectral profiles of the analyzed signal and idler beams. The central points $({k}_{{\rm{s}}}^{0\perp },{\omega }_{{\rm{s}}}^{0})$ and $({k}_{{\rm{i}}}^{0\perp },{\omega }_{{\rm{i}}}^{0})$ are positioned symmetrically at the phase-matching curve drawn in Fig. 2(a) that gives the magnitudes of transverse wave vectors ${k}_{{\rm{s}}}^{\perp }({\omega }_{{\rm{s}}})={k}_{{\rm{i}}}^{\perp }({\omega }_{{\rm{p}}}^{0}-{\omega }_{{\rm{s}}})$ as a function of frequency *ω*_s_. A suitably chosen angle *α* [$\tan (\alpha )=d{k}_{{\rm{s}}}^{\perp }/d{\omega }_{{\rm{s}}}{|}_{{\omega }_{{\rm{s}}}^{0}}$] shared by both beams transforms the original variables $(\delta {k}_{a}^{\perp },\delta {\omega }_{a})\equiv ({k}_{a}^{\perp }-{k}_{a}^{0\perp },{\omega }_{a}-{\omega }_{a}^{0})$ into the new ones $(\delta {k}_{a}^{{\rm{r}}\perp },\delta {\omega }_{a}^{{\rm{r}}})$ as depicted in Fig. 2(b). The corresponding transformations are expressed as follows:

7 $$[\begin{array}{c}\delta {k}_{{\rm{s}}}^{{\rm{r}}\perp }\\ \delta {\omega }_{{\rm{s}}}^{{\rm{r}}}\end{array}]=[\begin{array}{cc}\cos (\alpha ) & -\sin (\alpha )\\ \sin (\alpha ) & \cos (\alpha )\end{array}]\,[\begin{array}{c}\delta {k}_{{\rm{s}}}^{\perp }\\ \delta {\omega }_{{\rm{s}}}\end{array}],\,[\begin{array}{c}\delta {k}_{{\rm{i}}}^{{\rm{r}}\perp }\\ \delta {\omega }_{{\rm{i}}}^{{\rm{r}}}\end{array}]=[\begin{array}{cc}\cos (\alpha ) & \sin (\alpha )\\ -\sin (\alpha ) & \cos (\alpha )\end{array}]\,[\begin{array}{c}\delta {k}_{{\rm{i}}}^{\perp }\\ \delta {\omega }_{{\rm{i}}}\end{array}]\mathrm{.}$$

The azimuthal two-photon amplitudes Φ_*m*_ written in the transformed variables can then be approximately replaced by their factorized forms ${\tilde{{\rm{\Phi }}}}_{m}$:

8 $${\tilde{{\rm{\Phi }}}}_{m}(\delta {k}_{{\rm{s}}}^{{\rm{r}}\perp },\delta {\omega }_{{\rm{s}}}^{{\rm{r}}},\delta {k}_{{\rm{i}}}^{{\rm{r}}\perp },\delta {\omega }_{{\rm{i}}}^{{\rm{r}}})=\frac{{\tilde{{\rm{\Phi }}}}_{k,m}(\delta {k}_{{\rm{s}}}^{{\rm{r}}\perp },\delta {k}_{{\rm{i}}}^{{\rm{r}}\perp }){\tilde{{\rm{\Phi }}}}_{\omega ,m}(\delta {\omega }_{{\rm{s}}}^{{\rm{r}}},\delta {\omega }_{{\rm{i}}}^{{\rm{r}}})}{{{\rm{\Phi }}}_{m}({k}_{{\rm{s}}}^{0\perp },{\omega }_{{\rm{s}}}^{0},{k}_{{\rm{i}}}^{0\perp },{\omega }_{{\rm{i}}}^{0})}\mathrm{.}$$

The subsequent Schmidt decompositions of functions ${\tilde{{\rm{\Phi }}}}_{k,m}(\delta {k}_{{\rm{s}}}^{{\rm{r}}\perp },\delta {k}_{{\rm{i}}}^{{\rm{r}}\perp })$ and ${\tilde{{\rm{\Phi }}}}_{\omega ,m}(\delta {\omega }_{{\rm{s}}}^{{\rm{r}}},\delta {\omega }_{{\rm{i}}}^{{\rm{r}}})$,

9 $${\tilde{{\rm{\Phi }}}}_{k,m}(\delta {k}_{{\rm{s}}}^{{\rm{r}}\perp },\delta {k}_{{\rm{i}}}^{{\rm{r}}\perp })=\sum _{l=0}^{\infty }{\lambda }_{ml}^{\perp }{\tilde{u}}_{{\rm{s}},ml}(\delta {k}_{{\rm{s}}}^{{\rm{r}}\perp }){\tilde{u}}_{{\rm{i}},ml}(\delta {k}_{{\rm{i}}}^{{\rm{r}}\perp }),$$

10 $${\tilde{{\rm{\Phi }}}}_{\omega ,m}(\delta {\omega }_{{\rm{s}}}^{{\rm{r}}},\delta {\omega }_{{\rm{i}}}^{{\rm{r}}})=\sum _{q=0}^{\infty }{\lambda }_{mq}^{\parallel }{\tilde{f}}_{{\rm{s}},mq}(\delta {\omega }_{{\rm{s}}}^{{\rm{r}}}){\tilde{f}}_{{\rm{i}},mq}(\delta {\omega }_{{\rm{i}}}^{{\rm{r}}}),$$

${\sum }_{l=0}^{\infty }{\lambda }_{ml}^{\perp 2}=1$ and ${\sum }_{q=0}^{\infty }{\lambda }_{mq}^{\parallel 2}=1$, then allow us to arrive at the complete decomposition of the general two-photon amplitude $\tilde{{\rm{\Phi }}}$ expressed in the transformed variables in the following ’factorized’ form:

11 $$\tilde{{\rm{\Phi }}}(\delta {k}_{{\rm{s}}}^{{\rm{r}}\perp },\delta {\omega }_{{\rm{s}}}^{{\rm{r}}},{\phi }_{{\rm{s}}},\delta {k}_{{\rm{i}}}^{{\rm{r}}\perp },\delta {\omega }_{{\rm{i}}}^{{\rm{r}}},{\phi }_{{\rm{i}}})={\rm{\Lambda }}\sum _{m=-\infty }^{\infty }\sum _{l,q=0}^{\infty }{\lambda }_{mlq}{\tilde{g}}_{{\rm{s}},mlq}(\delta {k}_{{\rm{s}}}^{{\rm{r}}\perp },\delta {\omega }_{{\rm{s}}}^{{\rm{r}}},{\phi }_{{\rm{s}}}){\tilde{g}}_{{\rm{i}},mlq}(\delta {k}_{{\rm{i}}}^{{\rm{r}}\perp },\delta {\omega }_{{\rm{i}}}^{{\rm{r}}},{\phi }_{{\rm{i}}}\mathrm{).}$$

In Eq. (11), the global Schmidt numbers *λ*_*mlq*_ are given as

12 $${\lambda }_{mlq}={\lambda }_{m}{\lambda }_{ml}^{\perp }{\lambda }_{mq}^{\parallel },$$

the azimuthal Schmidt number *λ*_*m*_ is determined along the formula

13 $${\lambda }_{m}^{2}{{\rm{\Lambda }}}^{2}=\int d{k}_{{\rm{s}}}^{\perp }\int d{\omega }_{{\rm{s}}}\int d{k}_{{\rm{i}}}^{\perp }\int d{\omega }_{{\rm{i}}}|{{\rm{\Phi }}}_{m}({k}_{{\rm{s}}}^{\perp },{\omega }_{{\rm{s}}},{k}_{{\rm{i}}}^{\perp },{\omega }_{{\rm{i}}}{)|}^{2}$$

and the real overall coupling constant Λ is set such that ${\sum }_{m=-\infty }^{\infty }{\lambda }_{m}^{2}=1$. Using the Schmidt functions ${\tilde{u}}_{a,ml}$ and ${\tilde{f}}_{a,mq}$ occurring in Eqs. (9) and (10), respectively, and those defined in the azimuthal angles *φ*_*a*_,

14 $${v}_{{\rm{s}},m}({\phi }_{{\rm{s}}})=\frac{\exp (im{\phi }_{{\rm{s}}})}{\sqrt{2\pi }},\,{v}_{{\rm{i}},m}({\phi }_{{\rm{i}}})=\frac{\exp (\,\,-\,im{\phi }_{{\rm{i}}})}{\sqrt{2\pi }},$$

the overall Schmidt functions ${\tilde{g}}_{a,mlq}$ introduced in Eq. (11) are expressed by the relation:

15 $${\tilde{g}}_{a,mlq}(\delta {k}_{a}^{{\rm{r}}\perp },\delta {\omega }_{a}^{{\rm{r}}},{\phi }_{a})={\tilde{u}}_{a,ml}(\delta {k}_{a}^{{\rm{r}}\perp }){\tilde{f}}_{a,mq}(\delta {\omega }_{a}^{{\rm{r}}}){v}_{a,m}({\phi }_{a}),\,a={\rm{s}},i\mathrm{.}$$

Replacing the original fields’ operators ${\hat{a}}_{a}({k}_{a}^{\perp },{\phi }_{a},{\omega }_{a})$ defined for the harmonic plane-wave modes by the fields’ operators ${\hat{a}}_{a,mlq}$ of the overall Schmidt modes *g*_*a*,*mlq*_ (expressed in the original variables ${k}_{a}^{\perp }$ and *ω*_*a*_),

16 $${\hat{a}}_{a}({k}_{a}^{\perp },{\phi }_{a},{\omega }_{a})=\sum _{m=-\infty }^{\infty }\sum _{l,q=0}^{\infty }{g}_{a,mlq}({k}_{a}^{\perp },{\omega }_{a},{\phi }_{a}){\hat{a}}_{a,mlq},\,a={\rm{s}},{\rm{i}},$$

we arrive at the following decomposition of the spectral electric-field operator amplitudes ${\hat{E}}_{a}^{(+)}({k}_{a}^{\perp },{\omega }_{a},{\phi }_{a})$, *a* = s, i, given in Eq. (2)^55^:

17 $${\hat{E}}_{a}^{(+)}({k}_{a}^{\perp },{\omega }_{a},{\phi }_{a})=i\sqrt{\frac{\hslash {\omega }_{a}^{2}}{2{\varepsilon }_{0}{c}^{2}{k}_{a}}}\sum _{m=-{\rm{\infty }}}^{{\rm{\infty }}}\sum _{l,q=0}^{{\rm{\infty }}}{g}_{a,mlq}({k}_{a}^{\perp },{\omega }_{a},{\phi }_{a}){\hat{a}}_{a,mlq}.$$

Considering an impinging classical strong pump beam that depletes during its propagation in the nonlinear medium and the signal- and idler-beam electric-field operator-amplitude decompositions written in Eq. (17), we can approximately replace the original momentum operator ${\hat{G}}_{{\rm{int}}}$ given in Eq. (1) by the following momentum operator ${\hat{G}}_{{\rm{int}}}^{{\rm{av}}}$^55^:

18 $${\hat{G}}_{{\rm{int}}}^{{\rm{av}}}(z)=i\hslash \tilde{K}\sum _{m=-\infty }^{\infty }\sum _{l,q=0}^{\infty }{A}_{{\rm{p}},mlq}(z){\hat{a}}_{{\rm{s}},mlq}^{\dagger }(z){\hat{a}}_{{\rm{i}},mlq}^{\dagger }(z)+{\rm{H}}\mathrm{.}{\rm{c}}\mathrm{.}$$

The overall coupling constant $\tilde{K}$ is given as $\tilde{K}={\rm{\Lambda }}/(L{\xi }_{{\rm{p}}}^{({\rm{n}})})$, where the overall pump-beam amplitude ${\xi }_{{\rm{p}}}^{({\rm{n}})}=\sqrt{P/(f\hslash {\omega }_{{\rm{p}}}^{0})}$ is determined for photon numbers. A pump-beam amplitude *A*_p,*mlq*_ occurring in Eq. (18) characterizes the pump mode associated with an (*mlq*)th signal and idler dual Schmidt mode. We assume that the overall number of pump photons present in the incident pump beam is distributed into these modes linearly proportionally to the squared overall Schmidt coefficients ${\lambda }_{mlq}^{2}$, i.e. the incident pump-beam amplitude ${A}_{{\rm{p}},mlq}^{{\mathscr{N}}}$ of mode *mlq* related to normal ordering of field’s operators is given as

19 $${A}_{{\rm{p}},mlq}^{{\mathscr{N}}}={\lambda }_{mlq}{\xi }_{{\rm{p}}}^{({\rm{n}})}\mathrm{.}$$

This approach follows from the perturbation solution of the Schrödinger equation and the position of the Schmidt coefficients in the decomposition of the two-photon amplitude Φ_si_. Approximative ortho-normality of the pump mode functions plays an important role in this approach.

As discussed above the obtained pump modes are not normalized to unity and their re-normalization refines the model^40^. The corresponding re-normalization of the coupling constant $\tilde{K}$ is obtained as follows: A polychromatic signal photon with averaged energy $\hslash {\omega }_{{\rm{s}}}^{0}$ and mode function ${g}_{{\rm{s}},mlq}({k}_{{\rm{s}}}^{\perp },{\omega }_{{\rm{s}}},{\phi }_{{\rm{s}}})$ when annihilated together with a polychromatic idler photon having averaged energy $\hslash {\omega }_{{\rm{i}}}^{0}$ and mode function ${g}_{{\rm{i}},mlq}({k}_{{\rm{i}}}^{\perp },{\omega }_{{\rm{i}}},{\phi }_{{\rm{i}}})$ creates a pump photon with averaged energy $\hslash ({\omega }_{{\rm{s}}}^{0}+{\omega }_{{\rm{i}}}^{0})$ and properly normalized mode function (with unit norm). Such pump-photon mode function is easily determined in the crystal output plane (polar coordinates *r*_*a*_ and *φ*_*a*_, *a* = p, s, i) and time domain:

20 $${g}_{{\rm{p}},mlq}^{rt}(r,t)={g}_{{\rm{s}},mlq}^{rt}(r,t,\phi ){g}_{{\rm{i}},mlq}^{rt}(r,t,\phi \mathrm{).}$$

In Eq. (20), the signal- and idler-beam mode functions ${g}_{a,mlq}^{rt}({r}_{a},{t}_{a},{\phi }_{a})$ are given for the rotationally symmetric beams according to the formula

21 $${g}_{a,mlq}^{rt}({r}_{a},{t}_{a},{\phi }_{a})={s}_{a,mlq}^{rt}({r}_{a},{t}_{a}){v}_{a,m}({\phi }_{a}),$$

where

22 $${s}_{a,mlq}^{rt}({r}_{a},{t}_{a})=\frac{{i}^{m}}{\sqrt{2\pi }}{\int }_{0}^{\infty }d{k}_{a}^{\perp }\sqrt{{k}_{a}^{\perp }}{\int }_{-\infty }^{\infty }d{\omega }_{a}{u}_{a,ml}({k}_{a}^{\perp },{\omega }_{a}){f}_{a,mq}({k}_{a}^{\perp },{\omega }_{a}){{\rm{J}}}_{m}({k}_{a}^{\perp }{r}_{a})\exp (\,\,-\,i{\omega }_{a}{t}_{a})$$

and J_*m*_ denotes an *m*th order Bessel function. The functions *u* and *f* found in Eq. (22) are just the functions $\tilde{u}$ and $\tilde{f}$ written in the original variables *k*^⊥^ and *ω*. Determining the norm *κ*_p,*mlq*_ of an (*mlq*)th pump mode as

23 $${\kappa }_{{\rm{p}},mlq}^{2}=\frac{1}{2\pi }{\int }_{0}^{\infty }d{r}_{{\rm{p}}}{r}_{{\rm{p}}}{\int }_{-\infty }^{\infty }d{t}_{{\rm{p}}}|{g}_{{\rm{p}},mlq}^{rt}({r}_{{\rm{p}}},{t}_{{\rm{p}}}{)|}^{2},$$

we express the suggested re-normalization by the following substitutions:

24 $$\tilde{K}\leftarrow {\kappa }_{{\rm{p}},mlq}\tilde{K},\,{A}_{{\rm{p}},mlq}^{{\mathscr{N}}}\leftarrow \frac{\kappa }{{\kappa }_{{\rm{p}},mlq}}{A}_{{\rm{p}},mlq}^{{\mathscr{N}}}\mathrm{.}$$

The constant $\kappa =\mathrm{1/}\sqrt{{\sum }_{mlq}{\lambda }_{mlq}^{2}/{\kappa }_{{\rm{p}},mlq}^{2}}$ guarantees the correct number of pump photons, as it can be verified by the perturbation solution.

If the rotation angle *α* [or more precisely the angle *α*′ that transforms the variables $\delta {k}_{a}^{\perp }$ and *δω*_*a*_/*c* in agreement with the transformation in Eq. (7)], that expresses the strength of coupling between the spatial and spectral variables, is small and we neglect the part of re-normalization associated with the beams transverse plane (for sufficiently wide beams), we arrive at the simplified formula

25 $${\kappa }_{{\rm{p}},mlq}^{2}={\int }_{-\infty }^{\infty }d{\omega }_{{\rm{p}}}{|{\int }_{-\infty }^{\infty }d{\omega }_{{\rm{s}}}{f}_{{\rm{s}},mq}({\omega }_{{\rm{s}}}){f}_{{\rm{i}},mq}({\omega }_{{\rm{p}}}-{\omega }_{{\rm{s}}})|}^{2}$$

that expresses the energy conservation law $\hslash ({\omega }_{{\rm{s}}}+{\omega }_{{\rm{i}}})\leftrightarrow \hslash {\omega }_{{\rm{p}}}$.

The evolution of the interacting beams governed by the momentum operator ${\hat{G}}_{{\rm{int}}}^{{\rm{av}}}(z)$ in Eq. (18) for parametric down-conversion with the incident vacuum signal and idler beams was found in ref.^39^ in the generalized parametric approximation. In this approximation, independent evolution of the interacting modes’ triplets is first treated classically (using symmetric ordering of fields’ operators) which provides the pump-mode amplitude *A*_p,*mlq*_ of an (*mlq*)th triplet along the crystal in the form:

26 $${A}_{{\rm{p}},mlq}(z^{\prime} )={A}_{mlq}^{{\rm{ps}}}\frac{{A}_{mlq}^{{\rm{p}}}\,\cosh (\tilde{K}{A}_{mlq}^{{\rm{ps}}}z^{\prime} )-{A}_{mlq}^{{\rm{ps}}}\,\sinh (\tilde{K}{A}_{mlq}^{{\rm{ps}}}z^{\prime} )}{{A}_{mlq}^{{\rm{ps}}}\,\cosh (\tilde{K}{A}_{mlq}^{{\rm{ps}}}z^{\prime} )-{A}_{mlq}^{{\rm{p}}}\,\sinh (\tilde{K}{A}_{mlq}^{{\rm{ps}}}z^{\prime} )},$$

*z*′ = *z* + *L*, ${A}_{mlq}^{{\rm{p}}}=\sqrt{{({A}_{mlq}^{{\rm{p}}{\mathscr{N}}})}^{2}+\mathrm{1/2}}$ and ${A}_{mlq}^{{\rm{ps}}}=\sqrt{{({A}_{mlq}^{{\rm{p}}{\mathscr{N}}})}^{2}+1}$. The classical solution of the nonlinear evolution in each mode *mlq* is periodic and the formula for amplitude *A*_p,*mlq*_(*z*′) written in Eq. (26) is directly applicable only for positions *z*′ smaller than *z*′_0,*mlq*_ at which reversion of the nonlinear evolution occurs,

27 $${z^{\prime} }_{\mathrm{0,}mlq}=\frac{1}{2\tilde{K}{A}_{mlq}^{{\rm{ps}}}}\,\mathrm{ln}[1+\frac{2{A}_{mlq}^{{\rm{ps}}}}{{A}_{mlq}^{{\rm{ps}}}+\mathrm{1/}\sqrt{2}}\frac{{A}_{mlq}^{{\rm{p}}}-\mathrm{1/}\sqrt{2}}{{A}_{mlq}^{{\rm{ps}}}-{A}_{mlq}^{{\rm{p}}}}]\mathrm{.}$$

Then, in the second step, the linear Heisenberg equations for the signal and idler fields’ operators that incorporate the classical pump-mode solution,

28 $$\frac{d{\hat{a}}_{{\rm{s}},mlq}(z^{\prime} )}{dz^{\prime} }={K}_{mlq}^{{\rm{p}}}(z^{\prime} ){\hat{a}}_{{\rm{i}},mlq}^{\dagger }(z^{\prime} ),\,\frac{d{\hat{a}}_{{\rm{i}},mlq}(z^{\prime} )}{dz^{\prime} }={K}_{mlq}^{{\rm{p}}}(z^{\prime} ){\hat{a}}_{{\rm{s}},mlq}^{\dagger }(z^{\prime} ),$$

${K}_{mlq}^{{\rm{p}}}(z^{\prime} )\equiv \tilde{K}{A}_{{\rm{p}},mlq}(z^{\prime} )$, are solved. The solution is obtained in the following general form:

29 $$\begin{array}{c}{\hat{a}}_{{\rm{s}},mlq}(z^{\prime} )={U}_{mlq}(z^{\prime} ){\hat{a}}_{{\rm{s}},mlq}(z^{\prime} =\mathrm{0)}+{V}_{mlq}(z^{\prime} ){\hat{a}}_{{\rm{i}},mlq}^{\dagger }(z^{\prime} =\mathrm{0),}\\ {\hat{a}}_{{\rm{i}},mlq}(z^{\prime} )={U}_{mlq}(z^{\prime} ){\hat{a}}_{{\rm{i}},mlq}(z^{\prime} =\mathrm{0)}+{V}_{mlq}(z^{\prime} ){\hat{a}}_{{\rm{s}},mlq}^{\dagger }(z^{\prime} =\mathrm{0).}\end{array}$$

For the pump-beam amplitude *A*_p,*mlq*_(*z*′) from Eq. (26) the functions *U*_*mlq*_(*z*′) and *V*_*mlq*_(*z*′) attain the form^39^:

30 $${U}_{mlq}(z^{\prime} )=\,\cosh \,[{\varphi }_{mlq}(z^{\prime} )],\,{V}_{mlq}(z^{\prime} )=\,\sinh \,[{\varphi }_{mlq}(z^{\prime} )]$$

and

31 $${\varphi }_{mlq}({z}^{{\rm{^{\prime} }}})=\mathop{K}\limits^{ \sim }{A}_{mlq}^{{\rm{p}}{\rm{s}}}{z}^{{\rm{^{\prime} }}}-\,{\rm{l}}{\rm{n}}[\frac{{A}_{mlq}^{{\rm{p}}{\rm{s}}}+{A}_{mlq}^{{\rm{p}}}}{2{A}_{mlq}^{{\rm{p}}{\rm{s}}}}+\frac{{A}_{mlq}^{{\rm{p}}{\rm{s}}}-{A}_{mlq}^{{\rm{p}}}}{2{A}_{mlq}^{{\rm{p}}{\rm{s}}}}\exp (2\mathop{K}\limits^{ \sim }{A}_{mlq}^{{\rm{p}}{\rm{s}}}{z}^{{\rm{^{\prime} }}})].$$

## Physical quantities characterizing twin beams

The overall mean number *N* of photon pairs and number *K* of modes constituting a TWB are the most important ’integral’ characteristics of TWBs, i.e. characteristics independent of the form of spatio-spectral mode functions. The mean number *N*_s_ of signal photons, that coincides with the mean number *N* of photon pairs, is given as

32 $${N}_{{\rm{s}}}={\int }_{0}^{2\pi }d{\phi }_{{\rm{s}}}{\int }_{0}^{\infty }d{k}_{{\rm{s}}}^{\perp }{\int }_{-\infty }^{\infty }d{\omega }_{{\rm{s}}}\langle {\hat{n}}_{{\rm{s}}}({k}_{{\rm{s}}}^{\perp },{\omega }_{{\rm{s}}},{\phi }_{{\rm{s}}};L)\rangle ;$$

${\hat{n}}_{a}({k}_{a}^{\perp },{\omega }_{a},{\phi }_{a};L)\equiv {\hat{a}}_{a}^{\dagger }({k}_{a}^{\perp },{\omega }_{a},{\phi }_{a};L){\hat{a}}_{a}({k}_{a}^{\perp },{\omega }_{a},{\phi }_{a};L)$ for *a* = s, i. In Eq. (32), symbol 〈〉 means quantum mechanical averaging. Substituting Eq. (29) into the formula for *N*_s_ we have

33 $${N}_{{\rm{s}}}=\sum _{mlq}{V}_{mlq}^{2};$$

*V*_*mlq*_ = *V*_*mlq*_(*L*).

The overall number *K*^*n*^ of modes describing the TWB can be determined, e.g., by the formula derived for a multi-mode thermal field^55,58^:

34 $${K}^{n}=\frac{{(\sum _{mlq}{V}_{mlq}^{2})}^{2}}{\sum _{mlq}{V}_{mlq}^{4}}.$$

As an interesting alternative, the number *K* of modes can also be obtained directly from the statistics of photon pairs, as suggested in ref.^55^:

35 $$K=\frac{{(\sum _{mlq}{U}_{mlq}^{2}{V}_{mlq}^{2})}^{2}}{\sum _{mlq}{U}_{mlq}^{4}{V}_{mlq}^{4}};$$

*U*_*mlq*_ = *U*_*mlq*_(*L*).

More detailed information about the TWB properties, which is in the center of attention in this article, is provided by *spatially and spectrally resolved correlation functions*, especially by intensity profiles and intensity auto- and cross-correlation functions. In the transverse wave-vector and spectral domain, the formula for (local) signal-beam intensity ${I}_{{\rm{s}}}({k}_{{\rm{s}}}^{\perp },{\omega }_{{\rm{s}}},{\phi }_{{\rm{s}}})$ defined as

36 $${I}_{{\rm{s}}}({k}_{{\rm{s}}}^{\perp },{\omega }_{{\rm{s}}},{\phi }_{{\rm{s}}})=\langle {\hat{n}}_{{\rm{s}}}({k}_{{\rm{s}}}^{\perp },{\omega }_{{\rm{s}}},{\phi }_{{\rm{s}}};L)\rangle /{k}_{{\rm{s}}}^{\perp }$$

attains the form

37 $${I}_{{\rm{s}}}({k}_{{\rm{s}}}^{\perp },{\omega }_{{\rm{s}}},{\phi }_{{\rm{s}}})=\sum _{mlq}|{\bar{g}}_{{\rm{s}},mlq}({k}_{{\rm{s}}}^{\perp },{\omega }_{{\rm{s}}},{\phi }_{{\rm{s}}}{)|}^{2}{V}_{mlq}^{2};$$

functions ${\bar{g}}_{{\rm{s}},mlq}({k}_{{\rm{s}}}^{\perp },{\omega }_{{\rm{s}}},{\phi }_{{\rm{s}}})={g}_{{\rm{s}},mlq}({k}_{{\rm{s}}}^{\perp },{\omega }_{{\rm{s}}},{\phi }_{{\rm{s}}})/\sqrt{{k}_{{\rm{s}}}^{\perp }}$ take into account the rotationally symmetric geometry.

Similarly, intensity auto- (*A*) and cross- (*C*) correlation functions are defined as

38 $${A}_{{\rm{s}}}({k}_{{\rm{s}}}^{\perp },{\omega }_{{\rm{s}}},{\phi }_{{\rm{s}}};{k}_{{\rm{s}}}^{{}^{{\rm{^{\prime} }}}\perp },{\omega }_{{\rm{s}}}^{{}^{{\rm{^{\prime} }}}},{\phi }_{{\rm{s}}}^{{}^{{\rm{^{\prime} }}}})=\langle {\mathscr{N}}:{\rm{\Delta }}{\hat{n}}_{{\rm{s}}}({k}_{{\rm{s}}}^{\perp },{\omega }_{{\rm{s}}},{\phi }_{{\rm{s}}};L){\rm{\Delta }}{\hat{n}}_{{\rm{s}}}({k}_{{\rm{s}}}^{{}^{{\rm{^{\prime} }}}\perp },{\omega }_{{\rm{s}}}^{{}^{{\rm{^{\prime} }}}},{\varphi }_{{\rm{s}}}^{{}^{{\rm{^{\prime} }}}};L):\rangle /({k}_{{\rm{s}}}^{\perp }{k}_{{\rm{s}}}^{{}^{{\rm{^{\prime} }}}\perp }),$$

39 $${C}_{{\rm{s}}}({k}_{{\rm{s}}}^{\perp },{\omega }_{{\rm{s}}},{\phi }_{{\rm{s}}};{k}_{{\rm{i}}}^{\perp },{\omega }_{{\rm{i}}},{\phi }_{{\rm{i}}})=\langle {\mathscr{N}}:{\rm{\Delta }}{\hat{n}}_{{\rm{s}}}({k}_{{\rm{s}}}^{\perp },{\omega }_{{\rm{s}}},{\phi }_{{\rm{s}}};L){\rm{\Delta }}{\hat{n}}_{{\rm{i}}}^{\dagger }({k}_{{\rm{i}}}^{\perp },{\omega }_{{\rm{i}}},{\phi }_{{\rm{i}}};L):\rangle /({k}_{{\rm{s}}}^{\perp }{k}_{{\rm{i}}}^{\perp }),$$

symbol ${\mathscr{N}}::$ normally orders fields’ operators and ${\rm{\Delta }}\hat{x}=\hat{x}-\langle \hat{x}\rangle$ denotes the fluctuation of operator $\hat{x}$. We note that, in parallel to the correlation functions in Eqs (38) and (39), the spectrally and spatially averaged intensity correlation functions can be defined and more easily determined (for details, see ref.^39^). Substituting the relations (16) and (29) into Eqs (38) and (39) we arrive at the formulas for intensity auto- and cross-correlation functions:

40 $${A}_{{\rm{s}}}({k}_{{\rm{s}}}^{\perp },{\omega }_{{\rm{s}}},{\phi }_{{\rm{s}}};{k}_{{\rm{s}}}^{^{\prime} \perp },{\omega ^{\prime} }_{{\rm{s}}},\phi {^{\prime} }_{{\rm{s}}})={|\sum _{mlq}{\bar{g}}_{{\rm{s}},mlq}^{\ast }({k}_{{\rm{s}}}^{\perp },{\omega }_{{\rm{s}}},{\phi }_{{\rm{s}}}){\bar{g}}_{{\rm{s}},mlq}({k}_{{\rm{s}}}^{^{\prime} \perp },{\omega }_{{\rm{s}}}^{^{\prime} },{\phi }_{{\rm{s}}}^{^{\prime} }){V}_{mlq}^{2}|}^{2},$$

41 $${C}_{{\rm{s}}}({k}_{{\rm{s}}}^{\perp },{\omega }_{{\rm{s}}},{\phi }_{{\rm{s}}};{k}_{{\rm{i}}}^{\perp },{\omega }_{{\rm{i}}},{\phi }_{{\rm{i}}})={|\sum _{mlq}{\bar{g}}_{{\rm{s}},mlq}({k}_{{\rm{s}}}^{\perp },{\omega }_{{\rm{s}}},{\phi }_{{\rm{s}}}){\bar{g}}_{{\rm{i}},mlq}({k}_{{\rm{i}}}^{\perp },{\omega }_{{\rm{i}}},{\phi }_{{\rm{i}}}){U}_{mlq}{V}_{mlq}|}^{2}\mathrm{.}$$

Temporal properties of a TWB observed in the far field belong to important experimental characteristics. They are quantified by the appropriate intensities and intensity auto- and cross-correlation functions that are determined by the formulas analogous to those written above. Instead of the mode functions ${g}_{a,mlq}({k}_{a}^{\perp },{\omega }_{a},{\phi }_{a})$ needed when evaluating expressions in Eqs. (37), (40) and (41), we use the mode functions ${g}_{a,mlq}^{kt}({k}_{a}^{\perp },{t}_{a},{\phi }_{a})$ defined in the transverse wave-vector and time domain:

42 $${g}_{a,mlq}^{kt}({k}_{a}^{\perp },{t}_{a},{\phi }_{a})={s}_{a,mlq}^{kt}({k}_{a}^{\perp },{t}_{a}){v}_{a,m}({\phi }_{a}),\,a={\rm{s}},{\rm{i}},$$

and

43 $${s}_{a,mlq}^{kt}({k}_{a}^{\perp },{t}_{a})=\frac{1}{\sqrt{2\pi }}{\int }_{-\infty }^{\infty }d{\omega }_{a}\,{u}_{a,ml}({k}_{a}^{\perp },{\omega }_{a}){f}_{a,mq}({k}_{a}^{\perp },{\omega }_{a})\exp (\,\,-\,i{\omega }_{a}{t}_{a}\mathrm{).}$$

## Twin-beam intensities, intensity profiles and numbers of internal modes

To reveal the behavior as well as coherence of TWBs with the increasing pump power we consider a typical experimental configuration based on non-collinear spectrally non-degenerate type-I down-conversion in a BBO crystal of length *L* = 4 mm. The crystal is pumped by a pump beam of radius *w*_p_ = 500 *μ*m, central wavelength ${\lambda }_{{\rm{p}}}^{0}=349$ nm, spectral width Δ*λ*_p_ = 1 nm (FWHM) [*τ*_p_ = 0.75 ps] and repetition rate *f* = 400 s^−1^ that propagates along the *z* axis. For the BBO cut angle *θ*_BBO_ = 34.1 deg, the signal beam at central wavelength ${\lambda }_{{\rm{s}}}^{0}=668$ nm propagates outside the crystal at the central radial angle ${\theta }_{{\rm{s}}}^{0}=2.99$ deg whereas the idler beam centered around the wavelength ${\lambda }_{{\rm{s}}}^{0}=731$ nm leaves the crystal at the central radial angle ${\theta }_{{\rm{i}}}^{0}=3.28$ deg. We note that similar experimental configurations tuned for spectrally degenerate non-collinear down-conversion were used in refs^36,38^ where the experimental generation of ultra-intense TWBs in the regime with pump depletion was investigated by measuring the spatially and spectrally averaged intensity correlation functions. According to these experimental investigations, ultra-intense TWBs are expected for pump powers in tens or hundreds of mW in similar configurations when usual nonlinear crystals are used. For nonlinear structures with higher effective *χ*^(2)^ nonlinearities, ultra-intense TWBs are reached for lower pump powers inversely proportional to the squared *χ*^(2)^ coefficient. We also note that the previous theoretical investigations of intense TWBs^39,40,55^ were performed for spectrally degenerate TWBs as a consequence of limitations of the used model. Contrary to this, we consider here spectrally non-degenerate TWBs to demonstrate the range of applicability of the developed model. We also provide the comparison with the previously analyzed degenerate cases. Below, we analyze different TWBs occurring at the output plane of a crystal with ä fixed length that are generated by pump beams with the increasing intensities. This corresponds to the way how the experimental investigation of TWBs is performed. As shown below, a TWB intensity increases with the increasing pump-beam intensity. The properties of TWBs consecutively obtained this way are similar to those reached when we allow a pump beam with given initial intensity to propagate through a sufficiently long crystal. We note that the solution of the nonlinear interaction for one modes’ triplet depends on the crystal length *L* via the quantity $\sqrt{P}L$ involving the pump power *P*^39^. Properties of such propagating TWB including its coherence have been discussed in Introduction and schematically depicted in Fig. 1.

### Twin-beam intensities and numbers of internal modes

We first analyze the behavior of ’integral’ parameters of TWBs. This gives us a general framework for detailed discussion of the behavior of correlation functions on one side. On the other side, the comparison of properties of the analyzed spectrally non-degenerate TWBs with those being spectrally degenerate^39^ allows us to elucidate the role of the difference of the (central) signal and idler frequencies in forming a TWB during the nonlinear dynamics.

**Figure 3:**
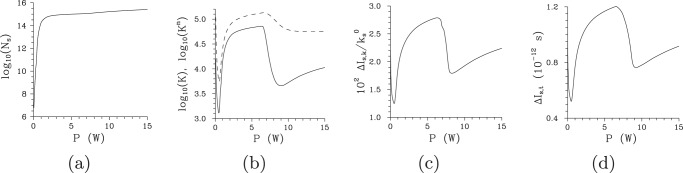
(**a**) Overall number *N*_*s*_ of signal photons, (**b**) numbers *K* (solid curve) and *K*^n^ (dashed curve) of modes given by Eqs. (35) and (34), respectively, and (**c**,**d**) widths Δ*I*_s,*k*_ (**c**) and Δ*I*_s,*t*_ (**d**) of the signal-beam intensity profiles in radial wave-vector direction and time, respectively, as they depend on pump power *P*. The widths are determined as the full widths at half maximum (FWHM) at the beam center, i.e. at ${\omega }_{{\rm{s}}}^{0}$ and ${k}_{{\rm{s}}}^{0\perp }$, respectively.

For our spectrally non-degenerate TWBs, the overall number *N* of photon pairs increases when the pump power *P* increases. Whereas this increase is exponential for lower pump powers (due to the properties of parametric down-conversion with an un-depleted pump beam), it gradually becomes linear for sufficiently high pump powers. As evident from the curve in Fig. 3(a) that gives the number *N*_s_ of signal photons as a function of pump power *P*, both areas are well separated. Explanation of this behavior was given in ref.^39^, in relation to the number *K* of effectively populated TWB modes: During the initial TWB amplification from the signal- and idler-beam vacuum state, the spatio-spectral modes with greater nonlinear coupling constants ${K}_{mlq}\equiv \tilde{K}{A}_{mlq}^{{\rm{ps}}}$ are amplified faster than the modes with lower coupling constants *K*_*mlq*_ and this behavior gradually suppresses the modes with lower coupling constants in the TWB. As a consequence, the number *K* of TWB modes decreases for lower pump powers *P*, until certain threshold pump power *P*_th_ is reached [see Fig. 3(b)]. For the pump powers *P* greater than the threshold power *P*_th_, the number *K* of modes again increases. The reason is that the TWB modes with the greatest coupling constants *K*_*mlq*_ are already at the stage of evolution in which they return back their energy to the corresponding pump mode. This diminishes the role of such modes in the structure of the TWB and it allows the modes with lower coupling constants ’to be seen’ again in the TWB. For the analyzed TWB, this threshold occurs around *P*_th_ = 250 mW and, as the curves for the numbers *K* and *K*^n^ of modes plotted in Fig. 3(b) show, there occurs also the second threshold around the power *P*_th,2_ ≈ 8 W^39^. This second threshold is observed at the pump powers for which the TWB modes with the greatest coupling constants, that loose their energy after the first threshold power *P*_th_ and later take back this energy, start again to loose their energy. We can see in Fig. 3(b) that the reduction of the number *K* of modes is smaller for the powers around *P*_th,2_ compared to that around *P*_th_ and, moreover, there occur two groups of populated modes with different values of their coupling constants in this case (for details, see ref.^39^). The presence of two groups of modes around *P*_th,2_ results in weakening of the effects of narrowing the TWB profiles and increasing the TWB coherence compared to those observed in the area around *P*_th_, as discussed below.

The reduction of the number *K* of TWB modes naturally leads to narrowing of TWB intensity profiles both in radial wave-vector and frequency domains [see Fig. 3(c) for the widths Δ*I*_s,*k*_ of signal-beam radial wave-vector intensity profiles]. This narrowing has its origin in the properties of the Schmidt modes defined in the (*k*^⊥^, *ω*) plane: The smaller the coupling constant of a modes’ triplet the wider the mode functions in the (*k*^⊥^, *ω*) plane (for typical intensity mode profiles, see ref.^59^). It holds both in the spectral and temporal domains, as well as in the transverse wave-vector domain, that the intensity profiles of a *q*th mode exhibit *q* + 1 local peaks and their overall widths increase with *q*. The reduction of the number of TWB modes thus causes *simultaneous narrowing of both spectral and temporal intensity profiles* for the pump powers around *P*_th_, as documented in Fig. 3(d). We note that this behavior, that stems from the nonlinearity of the analyzed interaction, qualitatively contrasts with the property of the (linear) Fourier transform that assigns longer pulses to narrower spectra.

The described behavior is qualitatively similar to that found for spectrally degenerate TWBs^39^. Thus, the spectral non-degeneracy, that qualitatively influences the properties of weak fields composed of individual photon pairs, plays only a minor role in forming the basic properties of ultra-intense TWBs. This is related to the fact that the dynamics of a TWB effectively evolves in greater number of orthogonal Schmidt spectral modes, each being spread over a large part of the spectrum.

### Twin-beam intensity profiles

**Figure 4:**
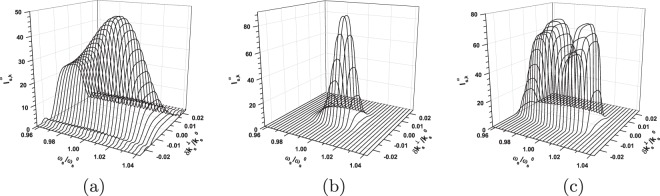
Signal-beam intensity profiles ${I}_{{\rm{s}},k}^{\omega }$ in radial wave-vector direction ${k}_{{\rm{s}}}^{\perp }$ for fixed signal frequencies *ω*_s_ for (**a**) P = 1 × 10^−8^ W, (**b**) *P* = 0.5 W and (**c**) *P* = 1.5 W. Centers of the drawn profiles are positioned along the axis $\delta {\omega }_{{\rm{s}}}^{{\rm{r}}}$, see Fig. 2(c). Profiles are normalized such that ${\int }_{0}^{\infty }d{k}_{{\rm{s}}}^{\perp }{I}_{{\rm{s}},k}^{\omega }({k}_{{\rm{s}}}^{\perp })/{k}_{{\rm{s}}}^{0\perp }=1$ for ${\omega }_{{\rm{s}}}={\omega }_{{\rm{s}}}^{0}$.

Now, we pay attention to the evolution of spatial, spectral and temporal TWB intensity profiles towards ultra-intense beams. To qualitatively discuss this evolution, we compare in Fig. 4 the shapes of spectrally resolved intensity profiles ${I}_{{\rm{s}},k}^{\omega }({k}_{{\rm{s}}}^{\perp })$ in the radial wave-vector direction of the signal beam for three specific cases. The shapes of a weak signal beam, as more-less formed by spontaneous parametric down-conversion, are plotted in Fig. 4(a). In the frequency domain and our configuration, the signal- and idler-beam spectra match each other: They together form a common spectrum of down-conversion observed in a given propagation direction. The shapes of intensity profiles ${I}_{{\rm{s}},k}^{\omega }({k}_{{\rm{s}}}^{\perp })$ drawn in Fig. 4(b) for a TWB around the threshold power *P*_th_ exhibit considerable narrowing in the whole (*k*^⊥^, *ω*) plane. These profiles broaden in the (*k*^⊥^, *ω*) plane for the pump powers *P* exceeding *P*_th_, as documented in Fig. 4(c) for a TWB generated for *P* = 1.5 W. Moreover and importantly, the spectral profiles attain a two-peak structure, which is a consequence of the inverted flow of energy in the modes with the greatest coupling constants. We note that the spectral modes with even mode numbers (*q* = 0, 2, …) have central peaks in their intensity profiles and greater coupling constants compared to their closest lower neighbors with odd mode numbers and central dips in their profiles. On the other hand, the intensity profiles ${I}_{{\rm{s}},k}^{\omega }({k}_{{\rm{s}}}^{\perp })$ in the radial wave-vector direction remain single peak.

**Figure 5:**
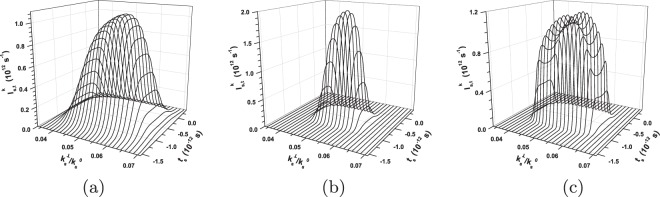
Signal-beam temporal intensity profiles ${I}_{{\rm{s}},t}^{k}$ for fixed signal radial wave vectors ${k}_{{\rm{s}}}^{\perp }$ for (**a**) *P* = 1 × 10^−8^ W, (**b**) *P* = 0.5 W and (**c**) *P* = 1.5 W. Centers of the drawn profiles are positioned along the axis $\delta {k}_{{\rm{s}}}^{{\rm{r}}\perp }$, see Fig. 2(c). Profiles are normalized such that ${\int }_{-\infty }^{\infty }d{t}_{{\rm{s}}}{I}_{{\rm{s}},t}^{k}({t}_{{\rm{s}}})=1$ for ${k}_{{\rm{s}}}^{\perp }={k}_{{\rm{s}}}^{0\perp }$.

The comparison of shapes of temporal intensity profiles ${I}_{{\rm{s}},t}^{k}({t}_{{\rm{s}}})$ resolved in the radial wave vector and plotted for the signal beam in Fig. 5 reveals qualitative similarity with the already discussed spectral shapes of Fig. 4. Also here, there occurs narrowing of the profiles around the threshold power *P*_th_ and two-peak structure of the temporal profiles for powers *P* exceeding *P*_th_. This two-peak structure is a consequence of the properties of temporal mode functions that are qualitatively similar to those of the spectral mode functions discussed above.

## Spatial, spectral and temporal coherence of twin beams, coherence waves

Now we come to the central results of our article discussing the behavior of spatial, spectral and temporal intensity auto- and cross-correlation functions in different areas of the TWB profiles as they evolve with the increasing pump power.

### Local coherence of twin beams as it depends on pump power, coherence waves

**Figure 6:**
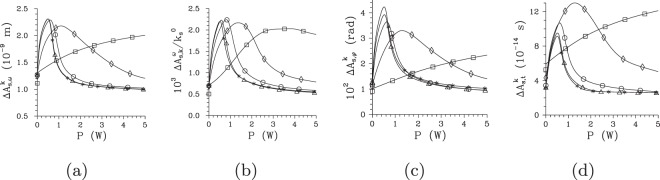
Widths ${\rm{\Delta }}{A}_{{\rm{s}},\omega }^{k}$ (**a**), ${\rm{\Delta }}{A}_{{\rm{s}},k}^{\omega }$ (**b**), ${\rm{\Delta }}{A}_{{\rm{s}},\phi }^{k}$ (**c**), and ${\rm{\Delta }}{A}_{{\rm{s}},t}^{k}$ (**d**) of the corresponding signal-beam intensity auto-correlation functions in in turn spectral, radial wave-vector, azimuthal wave-vector, and time variables [FWHM] as they depend on pump power *P*. In (**a**), (**c**) and (**d**) the reference points of the auto-correlation functions with coordinates $\delta {k}_{{\rm{s}}}^{\perp }/{k}_{{\rm{s}}}^{0}=0.0523$ (*), 0.0564 (Δ), 0.0605 (○), 0.0646 (◊), and 0.0728 (□) are positioned at the axis $\delta {k}_{{\rm{s}}}^{{\rm{r}}\perp }$; in (**b**) the reference points with coordinates $\delta {\omega }_{{\rm{s}}}/{\omega }_{{\rm{s}}}^{0}=0$ (*), 0.0085 (Δ), 0.0170 (○), 0.0254 (◊), and 0.0424 (□) lie at the axis $\delta {\omega }_{{\rm{s}}}^{{\rm{r}}}$ [see Fig. 2(c)].

The suppression of modes with larger mode numbers *m*, *l* and *q* (and thus smaller coupling constants) with the increasing pump power *P*, that narrows the intensity profiles, broadens the intensity auto- and cross-correlation functions. This can be understood by the fact that the complex mode functions belonging to the modes with small mode numbers *m*, *l* and *q* change slowly their phases along their intensity profiles. As follows from the comparison of curves in Fig. 6(a–d) showing the widths ${\rm{\Delta }}{A}_{{\rm{s}},\omega }^{k}$, ${\rm{\Delta }}{A}_{{\rm{s}},k}^{\omega }$, ${\rm{\Delta }}{A}_{{\rm{s}},\phi }^{k}$, and ${\rm{\Delta }}{A}_{{\rm{s}},t}^{k}$ of the signal-beam intensity auto-correlation functions in in turn frequency, radial wave-vector, azimuthal wave-vector, and time domain, this occurs more-less synchronously in all three directions of the signal-beam ’phase-space’ spanned by transverse wave vector and frequency, or time instead of frequency. According to the curves in Fig. 6(a–d), in the centers of intensity profiles the maxima of the widths of intensity auto-correlation functions are found around the threshold power *P*_th_. This accords with the results of ref.^39^ where the spatially and spectrally averaged intensity correlation functions were analyzed. However, the curves drawn in Fig. 6(a–d) reveal that these maxima gradually move towards the greater pump powers *P* when we move away from the centers of intensity profiles towards their edges. Even at the edges of the intensity profiles, the reached maximal widths of the intensity correlation functions are comparable to those observed at the centers of intensity profiles, but at greater pump powers. Moreover, these maxima occur for TWBs effectively composed of much larger numbers *K* of modes compared to that determined at the threshold power *P*_th_. This means that the improved coherence at the TWB edges is caused by some form of local phase synchronization of the Schmidt modes. This behavior forms *coherence waves* in the TWB ’phase-space’, as discussed in Introduction.

**Figure 7:**
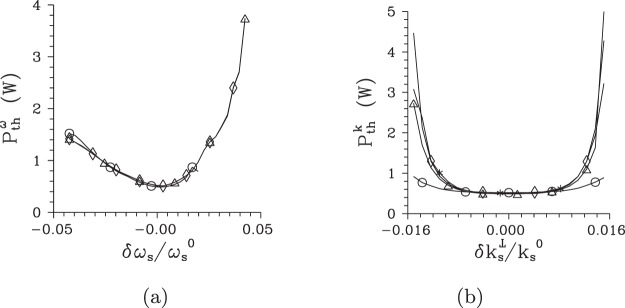
Threshold pump power ${P}_{{\rm{th}}}^{\omega }$ (**a**) [${P}_{{\rm{th}}}^{k}$ (**b**)] derived from maximal values of the widths of intensity auto-correlation functions expressed in signal radial wave vector (${\rm{\Delta }}{A}_{{\rm{s}},k}^{\omega }$, [${\rm{\Delta }}{A}_{{\rm{s}},k}^{k}$], Δ), signal frequency (${\rm{\Delta }}{A}_{{\rm{s}},\omega }^{\omega }$, [${\rm{\Delta }}{A}_{{\rm{s}},\omega }^{k}$], ○), signal azimuthal wave-vector angle (${\rm{\Delta }}{A}_{{\rm{s}},\phi }^{\omega }$, [${\rm{\Delta }}{A}_{{\rm{s}},\phi }^{k}$], ◊), and signal time ([${\rm{\Delta }}{A}_{{\rm{s}},t}^{k}$], *) and determined for points at the $\delta {\omega }_{{\rm{s}}}^{{\rm{r}}}$ [$\delta {k}_{{\rm{s}}}^{{\rm{r}}\perp }$] axis parameterized by *δω*_s_ [$\delta {k}_{{\rm{s}}}^{\perp }$].

To understand systematically the behavior of local threshold powers *P*_th_ in the whole signal-beam ’phase-space’, we determine their values from the behavior of intensity auto-correlation functions *A* computed along two orthogonal directions in the plane $({k}_{{\rm{s}}}^{\perp },{\omega }_{{\rm{s}}})$ that coincide with the axes $\delta {k}_{{\rm{s}}}^{{\rm{r}}\perp }$ and $\delta {\omega }_{{\rm{s}}}^{{\rm{r}}}$ of the rotated coordinate system [see Fig. 2(c)]. In each point in these directions, the local threshold powers *P*_th_ can be inferred from the correlation functions defined in three orthogonal directions, i.e. when we consider them as functions of radial wave vector (${k}_{{\rm{s}}}^{\perp }$), frequency (*ω*_s_) and azimuthal wave-vector angle (*φ*_s_). The curves plotted in Fig. 7(a,b) for the cuts along the $\delta {k}_{{\rm{s}}}^{{\rm{r}}\perp }$ and $\delta {\omega }_{{\rm{s}}}^{{\rm{r}}}$ axes show that the local threshold powers *P*_t*h*_ belonging to the correlation functions in three orthogonal directions are close to each other. This is true also for the local threshold power *P*_th_ derived from the temporal intensity auto-correlation functions ${A}_{{\rm{s}},t}^{k}$ at the $\delta {k}_{{\rm{s}}}^{{\rm{r}}\perp }$ axis, as evidenced in the curves of Fig. 7(b). We can roughly say that the smaller the local intensity *I*_s_ in a given point in the signal-beam ’phase-space’ the greater the local threshold power *P*_th_ (in all orthogonal directions). This suggests an idea that certain local Schmidt modes can be defined around an analyzed point in the TWB ’phase-space’ and the evolution of local coherence can be explained from the point of view of the evolution of populations of these local modes in the same vein as we did in the case of the whole TWB. This idea generalizes the approach that estimates the number of modes as the Fedorov ratio^60^, i.e. from the spread of local intensity correlation functions.

### Typical profiles of intensity auto- and cross-correlation functions

**Figure 8:**
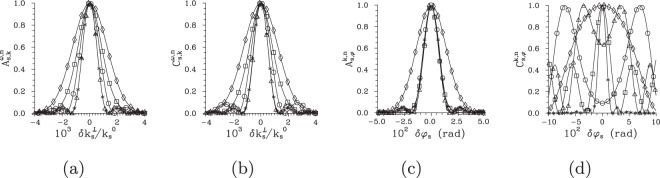
Profiles of normalized signal-beam intensity auto- [${A}_{{\rm{s}},k}^{\omega ,{\rm{n}}}$ (**a**), ${A}_{{\rm{s}},\phi }^{k,{\rm{n}}}$ (**c**)] and cross- [${C}_{{\rm{s}},k}^{\omega ,{\rm{n}}}$ (**b**), ${C}_{{\rm{s}},\phi }^{k,{\rm{n}}}$ (**d**)] correlation functions as they depend on normalized signal radial wave vector $\delta {k}_{{\rm{s}}}^{\perp }/{k}_{{\rm{s}}}^{0}$ (a,b) and signal azimuthal wave-vector angle *φ*_s_ (**c**,**d**). In (**a**) [(**c**)], the reference points for auto-correlation functions *A* lie on the $\delta {\omega }_{{\rm{s}}}^{{\rm{r}}}$ [$\delta {k}_{{\rm{s}}}^{{\rm{r}}\perp }$] axis at the positions specified in the caption to Fig. 6. In (**b**) [(**d**)], the reference points for cross-correlation functions *C* in the idler beam correspond to those in the signal beam specified in (**a**) [(c)] in variables *k*^⊥^ and *ω* and *φ*_i_ = *φ*_s_ + *π*; *P* = 1.5 W.

Before we conclude this section, we discuss typical profiles of the intensity auto- and cross-correlation functions that have been analyzed above. It holds that the averaged intensity auto-correlation functions *A* in the transverse wave-vector and frequency domains are broader than their cross-correlation counterparts *C* for low pump powers *P* and they tend to coincide for greater powers *P*^39^. This behavior is observed also for the investigated *not-averaged* intensity auto- and cross-correlation functions in the areas of TWB ’phase-space’ where the local phase-matching conditions are close to the ideal ones found in the central part of the TWB. We note that the ideal phase-matching conditions occur in the ‘phase-space’ at positions $({k}_{{\rm{s}}}^{0\perp },{\omega }_{{\rm{s}}}^{0},{\phi }_{{\rm{s}}}=\phi )$ and $({k}_{{\rm{i}}}^{0\perp },{\omega }_{{\rm{i}}}^{0},{\phi }_{{\rm{i}}}=\phi +\pi )$ and parameter *φ* varies from 0 to 2*π*. Under these conditions, the intensity correlation functions form either a single smooth peak or small oscillations may be observed at the shoulders of this peak, as documented in Fig. 8(a,b) for the normalized radial wave-vector intensity auto- (${A}_{{\rm{s}},k}^{\omega ,{\rm{n}}}$) and cross- (${C}_{{\rm{s}},k}^{\omega ,{\rm{n}}}$) correlation functions. However, if there occurs certain local phase mismatch in the investigated ’phase-space’ point, the relation between the auto- and cross-correlation functions qualitatively changes. The cross-correlation functions, that are strongly sensitive to the local phase mismatch, shift their maxima into a different than expected point in the ’phase-space’ where better phase-matching conditions are found. In this case, the cross-correlation functions *C* are considerably broader than the corresponding auto-correlation functions *A*. The normalized azimuthal intensity cross-correlation functions ${C}_{{\rm{s}},\phi }^{k,{\rm{n}}}$ together with their much narrower azimuthal auto-correlation counterparts ${A}_{{\rm{s}},\phi }^{k,{\rm{n}}}$ plotted in Fig. 8(c,d) for the points outside the TWB central part may serve as an example. In this case, some of the cross-correlation intensity profiles ${C}_{{\rm{s}},\phi }^{k,{\rm{n}}}$ have a two-peak structure.

## Twin-beam coherence and density of modes

In this section, we analyze the coherence of three typical TWBs with different intensities and elucidate the relation between their coherence and densities of modes.

### Twin-beam coherence

**Figure 9:**
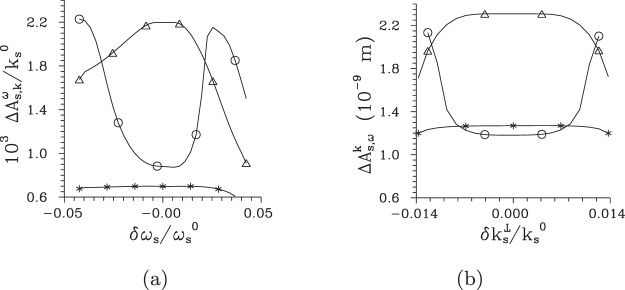
Widths ${\rm{\Delta }}{A}_{{\rm{s}},k}^{\omega }$ (**a**) [${\rm{\Delta }}{A}_{{\rm{s}},\omega }^{k}$ (**b**)] of signal-beam intensity auto-correlation functions in radial wave-vector [frequency] direction determined for points at the $\delta {\omega }_{{\rm{s}}}^{{\rm{r}}}$ [$\delta {k}_{{\rm{s}}}^{{\rm{r}}\perp }$] axis parameterized by *δω*_s_ [$\delta {k}_{{\rm{s}}}^{\perp }$] for *P* = 1 × 10^−8^ W (*), 0.5 W (Δ) and 1.5 W (○).

We consider a weak TWB (*P* = 1 × 10^−8^ W), a TWB generated around the threshold power (*P* = 0.5 W) and a TWB for which *P* = 1.5 W and mutually compare their properties. Similarly as above, we analyze the TWB coherence along the axes $\delta {k}_{{\rm{s}}}^{{\rm{r}}\perp }$ and $\delta {\omega }_{{\rm{s}}}^{{\rm{r}}}$ of the rotated coordinate system. As examples, the widths ${\rm{\Delta }}{A}_{{\rm{s}},k}^{\omega }$ and ${\rm{\Delta }}{A}_{{\rm{s}},\omega }^{k}$ of signal-beam intensity auto-correlation functions given in radial wave-vector and frequency domains, respectively, are drawn in Fig. 9(a,b) for three considered TWBs.

Whereas the signal-beam coherence is more-less uniform in the signal-beam ’phase-space’ for a weak TWB, it considerably varies in the ’phase-space’ of more intense TWBs. The analyzed more-intense TWBs generated with the pump powers 0.5 W and 1.5 W represent in certain sense two boundary cases. Whereas the maximal coherence in the less-intense TWB is observed in the center of its signal- and idler-beam profiles, the maximal coherence is reached at the edges of the profiles of the more-intense TWB.

**Figure 10:**
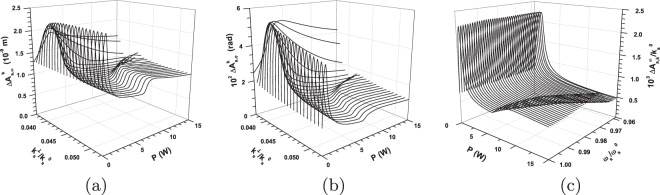
Widths ${\rm{\Delta }}{A}_{{\rm{s}},\omega }^{k}$ (**a**), ${\rm{\Delta }}{A}_{{\rm{s}},\phi }^{k}$ (**b**) and ${\rm{\Delta }}{A}_{{\rm{s}},k}^{\omega }$ (**c**) of the signal-beam intensity auto-correlation functions in in turn spectral, azimuthal wave-vector and radial wave-vector directions [FWHM] as they depend on pump power *P*. In (**a**) and (**b**) [(c)], the widths are drawn for fixed signal radial wave vectors ${k}_{{\rm{s}}}^{\perp }$ [frequencies *ω*_s_] giving the positions on the axis $\delta {k}_{{\rm{s}}}^{{\rm{r}}\perp }$ [$\delta {\omega }_{{\rm{s}}}^{{\rm{r}}}$] and scanning the lower [left-hand-side] half of the signal-beam radial wave-vector [frequency] intensity profile. For the coordinate system, see Fig. 2(c).

When we consider the TWBs with powers *P* increasing from the power *P* = 0.5 W to the power *P* = 1.5 W their area of maximal coherence moves from the signal- and idler-beam centers towards their edges. This is documented in Fig. 10 where the widths ${\rm{\Delta }}{A}_{{\rm{s}},\omega }^{k}$, ${\rm{\Delta }}{A}_{{\rm{s}},\phi }^{k}$ and ${\rm{\Delta }}{A}_{{\rm{s}},k}^{\omega }$ of signal-beam intensity auto-correlation functions determined in turn in frequency, azimuthal wave-vector and radial wave-vector domains are plotted along the axes $\delta {k}_{{\rm{s}}}^{{\rm{r}}\perp }$ and $\delta {\omega }_{{\rm{s}}}^{{\rm{r}}}$ as functions of pump power *P*. For the TWBs generated by even greater pump powers *P* > 1.5 W, the area of maximal coherence being at the edges of the TWB with *P* = 1.5 W moves further away from the beam center and disappears in the tails of beam profiles for sufficiently large powers *P* (see Fig. 10). As follows from the graphs in Fig. 10, qualitatively the same behavior of TWB coherence as observed for pump powers around the first threshold power *P*_th_ is found for the pump powers around the second threshold power *P*_th,2_. Also here coherence maxima occur in the beam center for the pump powers *P* below the threshold power *P*_th,2_ and they gradually move away from the beam center as the pump power *P* increases for *P* > *P*_th,2_. We can speak about *coherence waves* describing the propagation of coherence maxima across the beam profiles. The first two coherence waves are observed for the pump powers *P* > *P*_th_ and *P* > *P*_th,2_. The coherence waves propagate in the plane spanned by the signal (as well as idler) transverse wave-vector and frequency axes. The propagation of these waves reveals a detailed structure (origin) of coherence oscillations found along the power axis *P* for the averaged intensity correlation functions in ref.^39^.

### Densities of twin-beam modes

The change of coherence across a TWB profile can be interpreted as the change of the local densities *K*_*a*_ of modes, *a* = s, i, defined as the ratio of the volume in ’phase-space’ occupied by beam *a* and the local coherence volume:

44 $${K}_{a}={\mathscr{K}}\frac{2\pi {\rm{\Delta }}{I}_{a,k}{\rm{\Delta }}{I}_{a,\omega }}{{\rm{\Delta }}{A}_{a,k}{\rm{\Delta }}{A}_{a,\omega }{\rm{\Delta }}{A}_{a,\phi }}$$

**Figure 11:**
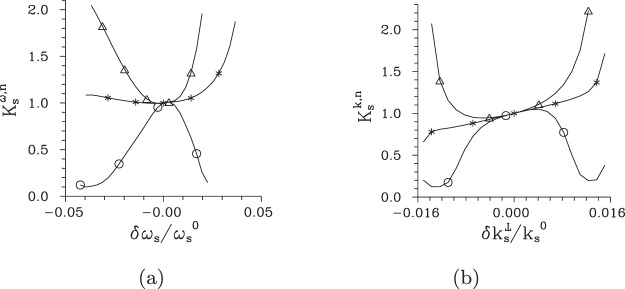
Normalized densities ${K}_{{\rm{s}}}^{\omega ,{\rm{n}}}$ (**a**) [${K}_{{\rm{s}}}^{k,{\rm{n}}}$ (**b**)] of modes determined for points at the $\delta {\omega }_{{\rm{s}}}^{{\rm{r}}}$ [$\delta {k}_{{\rm{s}}}^{{\rm{r}}\perp }$] axis parameterized by *δω*_s_ [$\delta {k}_{{\rm{s}}}^{\perp }$] for *P* = 1 × 10^−8^ W (*), 0.5 W (Δ) and 1.5 W (○).

In Eq. (44), the widths Δ*I*_*a*_ of intensity profiles are taken in the center of beam *a* at $({k}_{a}^{0\perp },{\omega }_{a}^{0})$ and the normalization constant *K* should be set such that the density *K*_*a*_ integrated over the whole beam profile gives the number ${\mathscr{K}}$ of modes determined by Eq. (35). According to the formula (44) the better the local beam coherence the smaller the local beam density *K*_*a*_ of modes. Thus, in agreement with the observation of the previous subsection, close-to-uniform densities of modes characterize weak TWBs. Intense nonlinear interaction then introduces changes into the densities of modes first reducing them in the central part of a TWB. For pump powers obeying *P* > *P*_th_, increase of the TWB intensity causes gradual movement of the area with the reduced densities of modes towards the edges of the TWB profile and then this area disappears in the TWB profile tails. Similar scenario is observed for greater pump powers *P* comparable or larger than the second threshold power *P*_th,2_. This means that there exist *waves in the densities of modes* that are synchronized with those in the TWB coherence. To illustrate this behavior we plot in Fig. 11(a,b) the normalized signal-beam densities ${K}_{{\rm{s}}}^{{\rm{n}}}$ of modes for the cuts along the axes $\delta {k}_{{\rm{s}}}^{{\rm{r}}\perp }$ and $\delta {\omega }_{{\rm{s}}}^{{\rm{r}}}$ of the rotated coordinate system. We remark that the normalized densities ${K}_{{\rm{s}}}^{k,{\rm{n}}}$ of modes considered as a function of radial transverse wave vector ${k}_{{\rm{s}}}^{\perp }$ in Fig. 11(b) show systematic increase of their values with the increasing ${k}_{{\rm{s}}}^{\perp }$. This reflects the rotational symmetry of the TWB that requires greater numbers of azimuthal modes for larger values of ${k}_{{\rm{s}}}^{\perp }$.

## Coherence of a twin beam propagating along the crystal

Before we conclude, let us rephrase the above findings obtained for TWBs generated from a crystal of fixed length by varying the pump power in terms of the development of the coherence of a TWB during its propagation in a sufficiently-long crystal. Considering, e.g., the signal beam in its ’phase-space’ spanned by the transverse wave vector and frequency (plotted along the propagation *z* axis), its shape resembles a ’blurred’ torus (rotationally symmetric around the *z* axis) with certain radius at low pump powers. This torus is gradually deflating as the signal beam propagates through the crystal, attains its minimal volume, then it is inflating until certain position in the crystal is reached. At this position it starts to deflate again. Starting from the input crystal face the TWB coherence increases inside the whole torus, the area of maximal coherence lies in the center of the torus. At the position where the volume of the torus is minimal (corresponding to the first threshold power *P*_th_), the area of maximal coherence starts to move towards the surface of the torus. It crosses the surface of torus in the area inside the crystal where the volume of torus is already large, continues its propagation outside the torus and gradually disappears. This represents *the first coherence wave*. Later in the crystal, similar behavior occurs. The area with coherence maxima is again formed in the center of the torus, but at certain position in the crystal (corresponding to the second threshold power *P*_th,2_) it starts to move towards the surface of torus forming *the second coherence wave*. Specific stages in the signal-beam evolution are depicted in Fig. 1 considering two 2D cuts of the torus by hyper-planes $({k}_{{\rm{s}}}^{\perp },{\phi }_{{\rm{s}}})$ and (*ω*_s_, *φ*_s_).

## Conclusions

We have predicted spatial, spectral and temporal waves in the coherence of ultra-intense twin beams during their evolution in the nonlinear interaction. Our prediction is based upon the analysis of a three-dimensional model of an intense rotationally-symmetric twin beam that was developed in the transverse wave-vector and frequency domains and then extended to the time domain. The model is based on the Schmidt decomposition of two-photon spatio-spectral amplitude. It treats the nonlinear evolution of parametric down-conversion in terms of suitable approximately independent modes’ triplets in the regime with pump-beam depletion. Intensity profiles of twin beams were determined as they change with the increasing pump power in a crystal of fixed length together with the intensity auto- and cross-correlation functions that vary across the twin-beam profiles. It was revealed that the maximal coherence of a twin beam is observed in the twin-beam central part provided that the generating pump power is lower than the threshold power. For greater pump powers above the threshold power, the rotationally-symmetric area of maximal coherence moves from the center of the signal (or idler) beam profile towards its edges as the pump power grows. These two-dimensional coherence waves across the signal (as well as the idler) beam profile drawn in the radial wave-vector and frequency plane are accompanied by two-dimensional waves in the local density of twin-beam modes. We believe that the predicted waves in the spatial, spectral, and temporal coherence of ultra-intense twin beams and the provided explanation will stimulate further theoretical as well as experimental investigations of the coherence of intense optical beams and open the door for applications of such beams, e.g., in metrology.

## Data Availability

All data generated and analyzed during this study are included in this published article.
